# Low RIN Value for RNA-Seq Library Construction from Long-Term Stored Seeds: A Case Study of Barley Seeds

**DOI:** 10.3390/genes11101190

**Published:** 2020-10-13

**Authors:** Marta Puchta, Maja Boczkowska, Jolanta Groszyk

**Affiliations:** National Centre for Plant Genetic Resources, Plant Breeding and Acclimatization National Research Institute, Radzików, 05-870 Błonie, Poland; m.puchta@ihar.edu.pl (M.P.); j.groszyk@ihar.edu.pl (J.G.)

**Keywords:** RNA-Seq, seed storage, RNA integrity number (RIN), *Hordeum vulgare*, degradation, mRNA stability, seed ageing

## Abstract

Seed aging is a complex biological process and its fundamentals and mechanisms have not yet been fully recognized. This is a key issue faced by research teams involved in the collection and storage of plant genetic resources in gene banks every day. Transcriptomic changes associated with seed aging in the dry state have barely been studied. The aim of the study was to develop an efficient protocol for construction of RNA-Seq libraries from long-term stored seeds with very low viability and low RNA integrity number (RIN). Here, barley seeds that have almost completely lost their viability as a result of long-term storage were used. As a control, fully viable seeds obtained in the course of field regeneration were used. The effectiveness of protocols dedicated to RNA samples with high and low RIN values was compared. The experiment concluded that library construction from low viable or long-term stored seeds with degraded RNA (RIN < 3) should be carried out with extraordinary attention due to the possibility of uneven degradation of different RNA fractions.

## 1. Introduction

Seed aging, seed deterioration or seed senescence has been defined as the loss of quality over time [1]. The most noticeable signs of seed aging at present are the increasing percentage of individuals in the seed lot that do not germinate (viability reduction) or germinate slowly (vigor reduction) over time. Seed aging is a complex biological feature in which several interrelated molecular, biochemical, physiological and metabolic processes are involved. All this makes the phenotype of seed longevity difficult to measure or predict, especially since it is also hard to assess, as the time before detectable changes occur is unknown. Walters et al. have shown that in a homogeneous seed lot there is usually a threshold at which individuals suddenly lose their viability [2]. In recent years, much effort has been put into research on seed aging processes, but the causes of seed senescence have still not been fully discovered. Furthermore, studies on the transcriptome in seeds that have been stored in a dry state and suffered loss of viability in the course of storage are very limited.

The transcriptome is a complete set of cell transcripts, characteristic for development or physiological state. Knowledge leading to understanding transcriptome activity is essential to interpret functional elements of the genome, reveal molecular components in cells and tissues and understand developmental processes [3].

The development of new high-throughput sequencing methods has provided an alternative method of mapping and quantification of transcripts [3]. RNA sequencing (RNA-Seq) uses the transcript profiling approach through deep sequencing and research and has changed the view on the complexity of transcripts. It provides a precise measurement of transcript expression level and their isoforms. The key aim of transcriptomic research is to catalogue different types of RNA molecules (mRNAs, sRNAs, non-coding RNAs) to determine the transcriptional structure of genes in relation to their starting point, splicing pattern and other posttranscriptional modifications to quantify changing expression levels of each transcript during development and under different environmental conditions. By contrast hybridisation, RNA-Seq is not limited to the detection of transcripts corresponding to a known genome sequence [3]. RNA-Seq has a very low, if any, existing background level compared to microarrays since DNA sequences can be unambiguously mapped to a unique genome region. RNA-Seq is an accurate method used to quantify the expression levels of transcripts as determined using RT-qPCR [4] and a spike-in RNA assay [5] with a known RNA concentration. It has high resolution and sensitivity, revealing many new transcribed regions and splicing isoforms of known genes, and allows mapping of exon and gene boundaries [6]. It enables absolute determination of the number of molecules in a cell population and comparison of the results between experiments. RNA-Seq analysis allows one to capture the dynamics of expression in different tissues under uncommon conditions without advanced data set normalization techniques [3]. An input for the construction of RNA-Seq libraries is the total RNA or individual fractions of e.g., mRNA, which is initially reverse transcribed to cDNA and subsequently ligated specific adaptors at one or both ends. Prepared libraries are amplified and sequenced from one end (single-end) or both ends (pair-end). Unlike sRNAs, which can be directly ligated to the adaptor, long RNA sequences have to be fragmented into smaller fragments ranging from 200 to 500 nt, which are compatible with the high throughput, next generation sequencing technology used in the experiment. Commonly used RNA fragmentation methods include hydrolysis or nebulization of RNA, while cDNA fragmentation is obtained by DNase I treatment and sonification [5].

A key aspect of RNAseq is to reduce the representation of redundant ribosomal RNA (rRNA) during the construction of libraries. rRNA is the most numerous fraction of total RNA, representing the majority (>80% to 90%) of molecules [7]. So far, several different solutions for reducing rRNA have been developed. In the literature, the following are most common: mRNA enrichment, ribosomal depletion and 3’mRNA counting. Probably the most widespread method used is poly(A)+ enrichment using oligo-dT coated beads performed prior to cDNA synthesis. However, the disadvantage of this method is elimination of non-rRNA populations, e.g., parts of non-coding RNAs. It may also introduce bias when analyzing low quality or low quantity RNA samples. The 3’mRNA counting method is based on reverse transcription using an oligo-dT primer [8]. After that the RNA template is removed and the second strand is synthesized using random primers [9]. The advantage of this method is that the direct number of readings reflects the number of transcripts of a given gene. cDNAs are only transcribed from the end of 3’RNA, and for each transcription only one copy of cDNA is generated [9]. The 3’ mRNA counting method requires well-annotated genomes and in addition it is not recommended for testing splicing sites [9,10]. It was observed that the RNA-Seq analysis identified 15% of transcripts with alternative splicing, while 3’ mRNA counting only identified 6% [9]. Another approach used in RNA-Seq libraries construction is rRNA depletion. There are currently three strategies for removing rRNAs. In the first one, rRNA is captured by complementary oligonucleotides coupled to paramagnetic beads and removed from the reaction. In the second method, rRNA is hybridized with oligos DNA and then RNA-DNA hybrids are degraded using RNase H. The third method involves transcribing total RNA on cDNA and preparing libraries with ZapR enzyme that targets ribosomal RNA sequences after conversion to cDNA [11]. Moreover, the rRNA depletion protocol may detect immature RNA (pre-RNA) apart from mature mRNA, while mRNA-Seq provides the highest sensitivity for detecting differences in gene expression, probably due to the larger fraction of readings mapped to the exome [12]. Zhao et al. [13] during research on blood and colorectal material showed that 220% and 50% more readings had to be sequenced using the rRNA depletion method to achieve the same level of exome coverage.

The most recent method is RNA capture that uses capture probes targeted at known exons for enriching coding RNA fractions. It allows sequencing transcripts by targeting them with an excess of probes at multiple positions, even if poly(A) tails were lost. Therefore, it is ideally suited for the analysis of degraded samples like those extracted from formalin-fixed, paraffin-embedded (FFPE) tissue samples [14]. However, this technique is used in human rather than plant samples. The rapid progress in the sequencing and annotation of plant genomes offers the prospect of extensive application of this technique for the analysis of plant samples, especially for major crops.

The aim of this study was to develop a methodological approach for the construction of RNA-Seq libraries from long-stored barley seeds with very low viability. Here, enrichment of the mRNA fraction using oligo-dT-coated beads was selected because a parallel analysis of the miRNA and degradome was conducted. Therefore, the results obtained from RNA-Seq and degradome-Seq will be compatible. The same method of reducing redundant rRNA will facilitate the interpretation of the results. To optimize the protocol of library construction from barley seeds with low viability, the SMARTer Stranded RNA-Seq Kit (Takara) was selected as it is dedicated to the analysis of non-mammalian samples. Moreover, it allows the use of both highly degraded and good quality RNA. This made it possible to compare the efficiency and correctness of the library construction protocol in an unbiased way.

The step of RNA-Seq library construction is crucial for further transcriptomic studies in seed aging and the results obtained here can be translated to other species.

## 2. Materials and Methods

### 2.1. Plant Material

**Figure 1:**
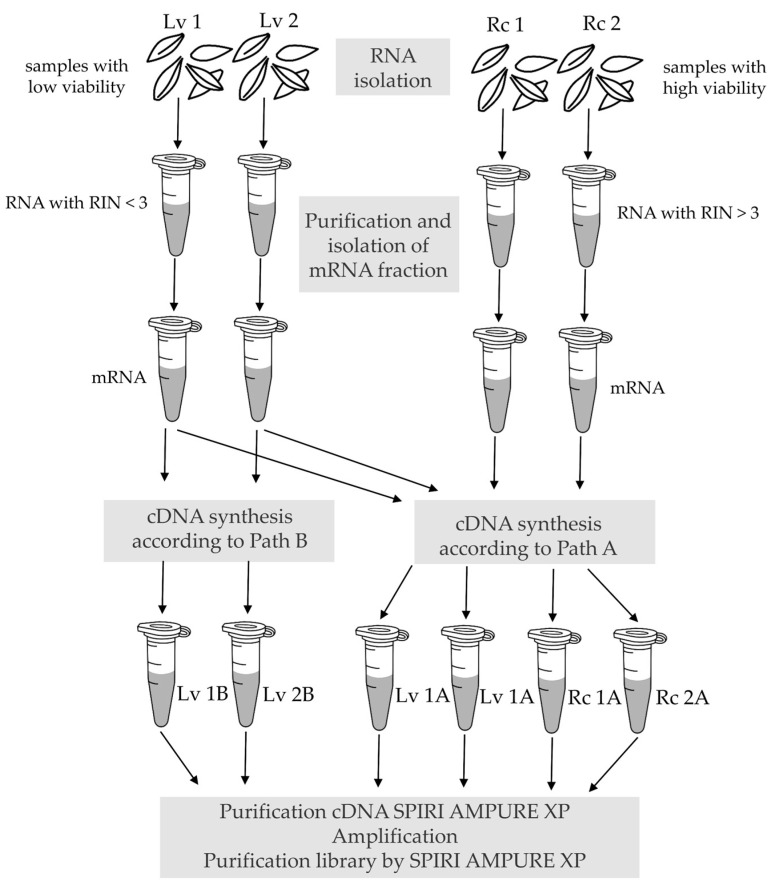
Diagram illustrating the experimental workflow (Rc—control samples with high viability, Lv—long-term stored samples with low viability; each sample represented by two biological replicates).

Experiment was carried out using grains from barley (*Hordeum vulgare* L.) cv. ‘Damazy’ harvested in 1972. The grains, which after harvest and drying showed above 95% viability and 2.96% moisture content, were placed in air-filled stocks, hermetically sealed and stored at room temperature. In 2015, the grain parameters were remeasured. The viability was 2%, moisture content 12.5%. Grains from the same initial lot with 99.3% viability (6.32% moisture content in 2015), preserved under gene bank conditions over the same period, were reproduced in a field trial in 2017 and used as reference sample. The grains obtained from the reproduction were dried in accordance with gene banks standards. Both grain samples, i.e., low viable (Lv) and reference sample (Rc), were used for RNA-Seq library preparation (Figure 1).

### 2.2. Total RNA Extraction

Total RNA was extracted from 25 dry embryos from each sample (Lv and Rc) in two biological replicates by crushing in liquid nitrogen and extracting using 1 mL of TRI Reagent (Sigma-Aldrich, Saint Louis, MO, USA) following the manufacturer’s protocol in the presence of 0.1 mL 1-bromo-3-chloropropane (Sigma-Aldrich, Saint Louis, MO, USA). The analyses were performed in two repetitions due to the limited number of low viable seeds. RNA pellets were washed in 1 mL of 75% ethanol (POCH, Gliwice, Poland) and after drying were dissolved in 100 µL of molecular grade water (A&A Biotechnology, Gdynia, Poland). The RNA was completely dissolved after 90 min in 4 °C, gently mixing every 30 min. The quantity and purity were assessed by a NanoDrop 1000 spectrophotometer (NanoDrop Technologies, Willmington, DA, USA ). Absorbance at 260 nm and 280 nm was used to assess the purity of nucleic acid samples. The quality of isolated total RNA was evaluated using the Bioanalyzer 2100 (Agilent, Santa Clara, CA, USA) by Eukaryote Total RNA Nano set (Agilent, Santa Clara, CA, USA).

### 2.3. Purification and Enrichment of the mRNA Fraction

Batches containing 100 µg of total RNA were purified from residual DNA using 10 µL dsDNase (Thermo Fisher Scientific, Waltham, MA, USA), 10 µL 10× dsDNase Buffer, and filled to the final 100 µL volume with molecular grade water. The samples were mixed and centrifuged and then incubated for 2 min at 37 °C in a preheated thermocycler (Applied Biosystem, Foster City, CA, USA). Total RNA after removing residual genomic DNA was used for mRNA enrichment using a Dynabeads Purification Kit for mRNA (Invitrogen, Carlsbad, CA, USA). For this purpose, 200 μL of Dynabeads was added to the sterile tube and placed in a magnetic stand for 30 s. In the next step, 80 µg of total RNA suspended in the volume of 100 μL was added to the beads (1:1 ratio) and mixed gradually for 5 min at room temperature. Beads with RNA attached to their surface were placed on a magnetic stand and 200 μL washing buffer was added after the supernatant was removed. The washing step was repeated twice. Beads were air-dried and eluted in 20 μL of sterile water. Beads were vortexed and then heated with mixing for 2 min at 65 °C and 500 rpm in a thermomixer (Biosan, Riga, Latvia). Afterwards, the tubes with beads were placed on a magnetic stand and 20 μL of supernatant containing enriched mRNA fraction was transferred to the pre-cooled tube.

### 2.4. cDNA Synthesis

In the next stage of library construction, cDNA synthesis was performed using a SMARTer Stranded RNA-Seq Kit (Takara, Kusatsu, Japan). According to the manufacturer’s recommendations, the cDNA synthesis protocol should depend on the quality of RNA measured by the RIN factor. For samples with RIN > 3, path A should be followed, while for degraded samples with low RIN < 3, path B is recommended. In pathway A, RNA is fragmented directly before cDNA synthesis by heat treatment in the presence of buffer. In pathway B, this step is omitted. Thus, samples with a low RIN value, i.e., originating from grains with low viability after long-term storage (Lv 1B and Lv 2B), were forwarded to pathway B. Analogously, samples with a high RIN value, i.e., isolated from non-stored seeds with a high viability (Rc 1A and Rc 2A), were directed to path A. Furthermore, samples with a low RIN value were also redirected to path A (Lv 1A and Lv 2A) (Figure 1).

#### 2.4.1. Path A for Samples with RIN > 3

Initially, 8 μL of mRNA (80 ng), 1 μL SMART Stranded N6 Primer (12 μM), and 4 μL 5× First Strand Buffer were used. Samples were incubated in a thermocycler for 5 min at 94 °C for RNA fragmentation and placed on ice for 2 min. Immediately afterwards, the remaining components were added: 0.5 μL DTT (100 mM), 0.5 μL RNase Inhibitor (40 U/μL), 2 μL dNTP Mix (10 mM), 2 μL SMARTer Stranded Oligo (12 μM), and 2 μL SMART Scribe RT (100 U/μL) in 20 μL total volume. The reaction mix was incubated in a thermocycler (Applied Biosystem, Foster City, CA, USA ) for 90 min at 42 °C, 10 min at 70 °C and cooled at 4 °C.

#### 2.4.2. Path B for Degraded Samples with RIN < 3

Initially, 8 μL of mRNA (80 ng) sample and 1 μL SMART Stranded N6 Primer (12 μM) were used. Samples were incubated in a thermocycler for 3 min at 72 °C and placed on ice for 2 min and the other components were subsequently added: 4 μL 5× First Strand Buffer, 0.5 μL DTT (100 mM), 0.5 μL RNase Inhibitor (40 U/μL), 2 μL dNTP Mix (10 mM), 2 μL SMARTer Stranded Oligo (12 μM), and 2 μL SMART Scribe RT (100 U/μL) in total volume of 20 μL. The sample was then incubated for 90 min at 42 °C, 10 min at 70 °C, and cooled at 4 °C.

### 2.5. Purification of cDNA using SPIRI AMPURE XP

All cDNA samples were purified using SPIRI AMPURE XP (Beckman Coulter, Brea, CA, USA) magnetic beads. After incubation, beads were vortexed for 30 min at room temperature and a volume of 20 μL was added to each sample, pipetted 10 times and incubated for 8 min at room temperature. In the next stage, each sample was placed on a magnetic stand for 5 min in order to precipitate the magnetic beads. Then the supernatant was removed and 200 μL of fresh 80% ethanol was added in order to wash the beads. After 30 s, the supernatant was removed and magnetic beads were washed in ethanol again. Pellets were dissolved in 22 μL of sterile water and used for amplification (cDNA solution contained SPIRI AMPURE XP beads).

### 2.6. Amplification

For amplification, 22 μL of purified cDNA was used. For each mix, 25 μL 2× SeqAmp PCR Buffer, 1 μL Universal Forward PCR Primer (12.5 μM), 1 μL Reverse PCR Primer (12.5 μM) different for each sample, and 1 μL SeqAmp DNA Polymerase were added. Samples were mixed and incubated in a thermocycler for 1 min at 94 °C followed by 10 cycles of: 15 s at 98 °C, 15 s at 55 °C, 30 s at 68 °C, and then cooled for 1 min at 4 °C.

### 2.7. Purification of cDNA Library by SPIRI AMPURE XP

Purification of cDNA library was performed again after the amplification, this time adding 50 μL of magnetic beads to each sample and the rest of the protocol as described above. The purified library was eluted in 20 μL of sterile water. Samples were incubated at room temperature for 2 min and thereafter placed again on a magnetic stand for several minutes. The supernatant was transferred to a new tube.

Prepared libraries were evaluated qualitatively and quantitatively using the Qubit fluorimeter (Thermo Fisher Scientific, Waltham, MA, USA) by Qubit dsDNA HS Assay Kit (Thermo Fisher Scientific, Waltham, MA, USA) and automatic electrophoresis system Bioanalyzer 2100 using High Sensitivity DNA kit (Agilent, Santa Clara, CA, USA).

### 2.8. NGS Sequencing

After normalization, the libraries were sequenced on a HiSeq 4000 machine (Illumina, San Diego, CA, USA) in paired-end 2× 150 bp. The RNA-Seq data results have been qualitatively evaluated using the FastQC software [15]. RNA libraries were sequenced by Genomed S. A. (Warsaw, Poland).

## 3. Results

### 3.1. RNA Quality

**Figure 2:**
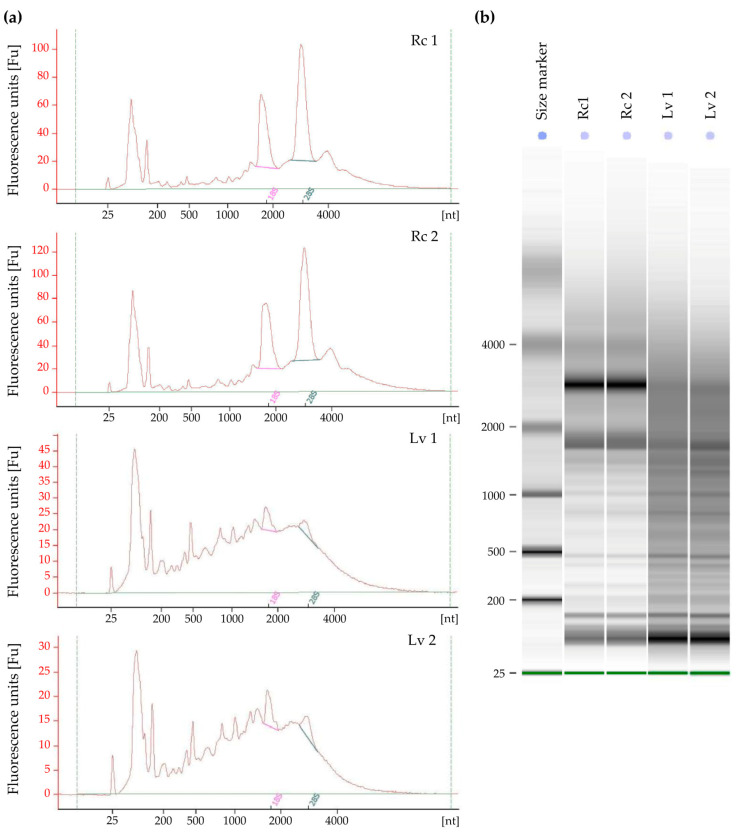
Results of total RNA extracted using the method described in this study. Extractions were made from four different samples (Rc—control samples with high viability, Lv—long-term stored samples with low viability; each sample represented by two biological replicates) and run on an Agilent 2100 Bioanalyzer using the Eukaryote Total RNA Nano kit (Agilent). (**a**) Agilent bioanalyzer chromatograms of total RNA; (**b**) Agilent bioanalyzer gel-like image of total RNA.

Isolated total RNA was evaluated qualitatively and quantitatively, according to the recommendations of the RNA-Seq library manufacturers for Next Generation Sequencing, the RIN (*RNA Integrity Number*) was measured. The analyses were performed for long-term stored barley grains with low viability and fresh grains after field reproduction with high viability. Due to the limited number of low viability grains, the analyses were performed in two biological replicates. According to the results, samples from fresh grains (Rc 1 and Rc 2) had good quality RNA and the RIN of the samples was 7.8 and 7.9, respectively (Figure 2). For samples from long-term stored grains (Lv 1, Lv 2), RNA showed a high grade of degradation, as indicated by RIN 2.5 and 2.8 (Table 1). The electropherograms in Figure 2 show a high grade of degradation of 18S and 28S rRNA subunits.

### 3.2. mRNA Enrichment

In the next step, as foreseen by the protocol, a dsDNase enzyme was used to remove contaminating DNA in RNA preparations. After decontamination, the sample concentration was about 900 ng/µL, which is about 45% of the initial samples for both low and high RNA, except for the Lv 2B sample, whose initial concentration was lower, and the recovery of the sample after dsDNase use was as high as 70%.

To prepare the RNA-Seq libraries, the mRNA fraction was enriched by oligo(dT) magnetic beads. After the reaction, the mRNA fraction was about 140 ng/µL in Rc 1 and Rc 2 samples, which was 13%, while for samples with low RIN, the concentration ranged from 62 ng/µL to 90 ng/µL (Table 1), which was up to 10% on average. The amounts of mRNA were consistent with the literature data [16].

### 3.3. Library Construction

**Figure 3:**
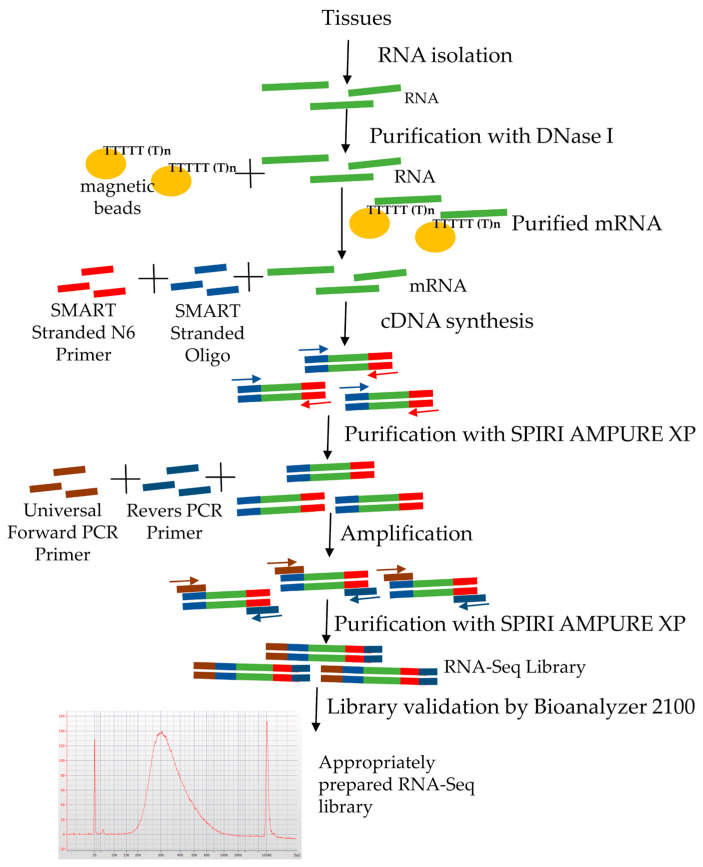
Stages of RNA-Seq library preparation.

The libraries were prepared according to the Takara protocol (SMARTer Stranded RNA-Seq) (Figure 3), which assumes two ways of preparing libraries depending on the RIN. For Rc 1 and Rc 2 samples with high RIN, cDNA synthesis was performed according to path A (Figure 1). The final library concentration prepared according to path A was 14.07 ng/µL (Rc 1) and 13.82 ng/µL (Rc 2), respectively (Table 2). Length distribution of fragments in prepared libraries was evaluated electrophoretically. The highest concentration was observed in the range from 250 to 600 bp. The highest distribution of Rc 1A fragments was observed for 259 bp and was 2038.59 ng/µL, while for Rc 2A it was 1906.00 ng/µL (Table S1).

For the Lv samples with low RIN, a cDNA synthesis was performed in accordance with path A (Lv 1A and Lv 2A) and path B (Lv 1B and Lv 2B). During electrophoretic analysis, the library was prepared according to path B, which is preferred for degraded total RNA, and the highest distribution of fragments was observed above 450 bp. The highest concentration for samples Lv 1B was observed for 880 bp fragments (concentration 115.08 ng/µL) and for Lv 2B 887 bp (concentration 130.23 ng/µL) (Figure 2). The final concentration of libraries constructed according to path B was 0.76 ng/µL and 0.82 ng/µL for Lv 1B and Lv 2B, respectively. The results indicate abnormal libraries, which is indicated by a high proportion of long DNA fragments and low final library concentrations. This indicates that mRNA integrity was not substantially decreased. However, for the construction of libraries for Lv samples according to path A for undegraded samples (omitting the low RIN value), the correct length distribution of DNA fragments in the libraries was observed. These results indicate that regardless of the low RIN, the appropriate electrophoretic RNA pattern was maintained and the mRNA is of good quality.

**Figure 4:**
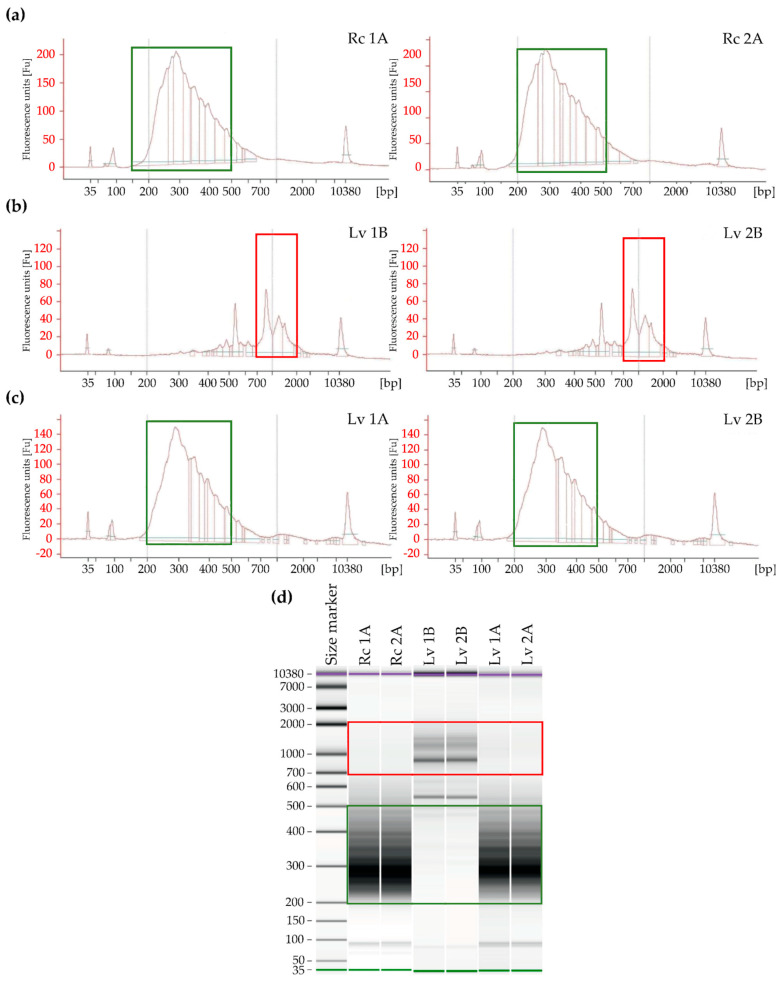
Densitometry validation and quantification of final RNA-Seq cDNA libraries prior to Illumina sequencing, using an Agilent 2100 Bioanalyzer and the High Sensitivity DNA kit (Beckman Coulter, Brea, CA, USA). The outermost peaks represent a size marker. RNA-Seq cDNA libraries were prepared from two replicates of RNA extracted from barley grains: (**a**) control samples with high viability according to the path A; (**b**) long-term stored samples with low viability according to the path B; (**c**) long-term stored samples with low viability according to the path A; (**d**) electrophoresis run of the cDNA libraries by the Bioanalyzer. The green and red frames indicate correct and incorrect fragment size range in the libraries, respectively.

The libraries had the highest concentration of fragments of 280 to 500 bp in length. The highest concentration in sample Lv 1A was observed for fragments of 288 bp length, which was 2138.73 ng/µL, and for samples Lv 2A of 286 bp, where the concentration was 1436.54 ng/µL. The constructed libraries had the final concentration in Lv-1A 5.38 ng/µL and in Lv 2A 5.4 ng/µL (Figure 4).

### 3.4. NGS Sequencing

As a result of Rc 1A, Rc 2A, Lv 1A and Lv 2A libraries sequencing, 28 to 39 million raw reads were obtained for each of them (Table 3). Due to an incorrect length range, the Lv 1B and Lv 2B libraries were not directed to sequencing. For each library, the per base sequence quality (Phred score) was nearly 40. The average percentage of GC content in each library was 55%. The sequenced fragments were in the range of 15–150 bp. Detailed information can be found in the supplementary files (Figures S1–S6).

## 4. Discussion

Overall, RNA integrity is the major factor affecting the quality of sequencing data [17]. Therefore, the protocols of RNA-Seq libraries require samples with high-quality RNA. Prior to the construction of the libraries, it is necessary to assess RNA integrity number (RIN), which has become a widely accepted standard for quality measurement and has proven to be more accurate than UV spectrophotometric measurements and pure ribosomal RNA ratios [18]. Electrophoretic techniques have been widely used to evaluate RNA degradation to separate molecules by size in the sample. Historically, integrity evaluation was performed using electrophoresis in an agarose gel stained with ethidium bromide, and usually, the gel showed two bands; 28S and 18S rRNA. In 1999, the Bioanalyzer Agilent 2100 was introduced to separate DNA, RNA, and protein samples [18]. This was an automated system, using micro-fluid technology that provides electrophoretic separation of samples in an automated and reproducible manner [19]. Small amounts of samples are separated in the micro-fabricated chip channels according to their molecular weight and then detected by laser detection. The result is an electropherogram in which the amount of changed fluorescence correlates with the amount of RNA of a given size. The software calculates the ratio of two ribosomal bands. The RIN measurement is based on a machine learning algorithm that uses a capillary electrophoresis pathway and not just on the ratio of ribosomal subunits, although it is highly dependent on it. It provides a numerical score (range 1–10) for RNA quality. A higher RIN value indicates a higher degree of RNA integrity [20]. It is generally accepted that for samples with high inter granularity, the RIN level should be about 7–8. Most commercially available RNA-Seq library preparation kits require a high RIN value of the input RNA for proper library construction. However, the measurement of RIN has several weaknesses, which include the RIN dependence on 18S and 28S ribosomal RNA, because ribosomal indicators have usually shown low correlation with RNA integrity [21]. Another disadvantage of the RIN technique is that it maps RNA integrity, but it does not directly measure mRNA integrity, which is the main genetic material used in the construction of the libraries [22]. On the other hand, the complexity of RNA analysis results in a large impact of the integrity and abundance of the input RNA on the quantification of transcripts, regardless of whether it is performed by qPCR, microarray or RNA-Seq [17,23,24]. Degraded samples during sequencing show an insufficient representation of long transcripts and overexpression of short transcripts [17].

RNA degradation is an integral part of cellular metabolism and regulation of gene expression [25,26]. mRNA molecules are mostly short-lived, their persistence varies from several hours to several days and usually results from posttranscriptional regulation of mRNA and targeted decomposition of damaged RNA [27,28]. It has been estimated that the half-life of typical mRNAs ranges from 1 to 30 h and that cell degradation progresses also after death [29]. However, transcripts in dead cells as well as degradation of an isolated RNA are not subjected to physiological processes occurring in a normally functioning cell. It is not clear whether the RNA degradation occurs randomly or is related to the specific properties of the transcript [30]. It was found that in most cases, given the results of RNA-Seq analyses, the most efficient approach includes all samples regardless of their quality [30]. Using RIN as the only quality assessment was questionable because RIN with a value of 2.5 and 2.8 for long-term stored barley seeds as well as 7.9 and 7.8 for reference samples was derived from ribosomal integrity and the process of degradation of mRNA transcripts may be species-specific, which is not necessarily reflected in RIN [31]. The expression of mRNAs was strictly regulated and transcripts were degraded at different rates by different mechanisms, partly in relation to their biological function [32]. According to Romero et al. [30], three approaches can be adopted to deal with reduced quality samples. First, samples with high RNA degradation could be rejected, but this could cause decrease the chance of obtaining unique results. Second, assuming that all types of transcripts disintegrate at a similar rate in respect to gene expression, RNA integrity can be estimated by using a standardization procedure. Third, the RIN value can also be assumed, if different transcripts decay at different rates, and these rates are consistent for a given level of RNA degradation [30]. The size distribution of undamaged RNA molecules is more diverse than DNA, therefore distinguishing large fragmented mRNA molecules from undamaged, smaller mRNA molecules is very difficult [33].

An interesting case is RNA in dry seeds, where transcripts persist even for decades until germination begins [34]. To survive, metabolic activity is switched off and the cytoplasm transforms into a glassy state in cells of the dry seed [35]. Proteins required for germination can be translated from stored mRNA or long-lived mRNA templates in the mature dry seeds [36,37]. Over 10,000 mRNA species were detected in mature dry seed of *Arabidopsis* [38]. It has been postulated that rapid restoration of seed metabolism during imbibition resulted from the early translation of stored mRNAs [39]. The integrity of RNA, as well as DNA, impacts on seed longevity. Germability decrease is accompanied by a reduction in total RNA content and RNA integrity [33,40,41]. Fleming et al. [42] demonstrated in soybean that longer transcripts were more likely to be damaged and postulated that this was due to the impact of reactive oxygen species. It has been proven that, during germination, the seeds translate stored mRNAs using stored ribosomes [43,44]. However, whether stored mRNAs are sufficient to allow the completion of germination may be species-specific, i.e., appearance of the radicle [36,37,45].

The results obtained in the study confirm RNA integrity loss expressed by lowering the RIN value resulting from the loss of the viability of barley grains due to ageing. However, due to the enrichment of samples with mRNA after isolation, the use of RIN appears to be inappropriate. Most RNA-Seq library construction kits are based on the enrichment of the mRNA fraction from the total RNA. This process is most often performed by using oligo(dT)-coated magnetic beads to capture the poly-adenylated end of mRNA and thereby discarding the problem of the highly abundant rRNA [46]. For seeds that have aged in a dry state, the degradation of different RNA fractions seems to occur at different rates. Major differences in RIN levels between samples with low germination capacity and fully viable samples were noted. However, as in Fleming et al. studies [33], they were not reflected by a substantial decrease in mRNA integrity. Using RIN values as an indicator of the degree of RNA degradation in such cases may lead to a library construction with inappropriate parameters, i.e., with an inaccurate distribution of fragment length. Treating samples with low RIN as highly degraded resulted in omitting the RNA fragmentation step during the construction of libraries, which resulted in a high concentration of overly long fragments inappropriate for correctly constructed libraries. According to the manufacturer’s recommendations, degraded samples should be treated according to path B, which skips the RNA fragmentation in the first stage. In the case of long-term stored barley grains, this is an incorrect approach because the libraries prepared in this way had the wrong length distribution of library fragments. However, it should be kept in mind that the majority of RNA-Seq library kits were designed to analyze human or mammalian samples. So the starting materials are often formalin-fixed or paraffin-embedded, which causes RNA degradation to a small average size [47]. According to the manufacturer, path A is intended only for samples with RIN < 3, but as we have shown in our research, it is wrong. Samples with low RIN with a proper RNA pattern should be cut in the first stage of the libraries construction. Using the protocol for high quality samples with RIN > 3, which includes the fragmentation step both for RNA samples obtained from low- and highly viable barley grains, allows one to get the correct concentration of fragments in the range of 250–600 bp. Although the protocol described above is based only on barley grains, we believe that it can also be successfully applied to seeds of other species stored in a dry state. A high grade of degradation of 18S and 28S rRNA subunits was also observed in RNA samples isolated from oat and rye grains, which substantially lost their viability due to long-term storage in a dry state [48]. Since rRNA depletion protocols were developed to overcome the problem of RNA degradation, libraries for RNA-Seq prepared in this way should also be treated as undegraded regardless of the RIN value for long-term stored seeds.

Many aspects like seed development, germination, viability, vigor or longevity have been investigated. Based on those results, our knowledge and understanding of aging processes and viability has progressed considerably [49,50,51,52]. Despite identifying so many mechanisms of seed aging, the genetic mechanisms underlying seed aging still remain to be discovered.

So far, the analyses of the transcriptome in stored seeds have been limited mainly to the termination of dormancy or accelerated artificial ageing of seeds due to short-term exposure to warm and humid conditions [41,53,54,55,56,57]. To the best of our knowledge, research on transcriptome changes related to ageing of dry stored seeds has been conducted only for soybean [33]. The analysis of transcriptome changes concerning the loss of viability during storage of seeds in a dry state is fundamental for gene banks where plant genetic resources are preserved for future.

## 5. Conclusions

Summarizing the results and the literature reports to date, the RIN value recommended by manufacturers of library construction kits does not indicate the level of degradation of the mRNA fraction in all cases. During the construction of transcriptome libraries from seeds with low viability after storage in a dry state and with low RIN, the assessment of the mRNA peak on the electropherography should be considered. The conducted tests indicate the presence of good quality mRNA in samples with low RIN. This indicates the need to apply the library design procedure for high RIN samples regardless of its value in order to obtain appropriate lengths and concentrations of cDNA fragments in the constructed libraries. The protocol and results presented here may provide an impulse to conduct transcriptomic research in samples with low RIN.

## Figures and Tables

**Table 1:** Sample parameters obtained by the automatic electrophoresis Bioanalyzer 2100 (Rc—control samples with high viability, Lv—long-term stored samples with low viability; each sample represented by two biological replicates).

	Samples	Rc 1	Rc 2	Lv 1	Lv 2
	RIN	7.9	7.8	2.5	2.8
	RNA Area	1437.8	1837.6	1253.1	820.5
	RNA Concentration [ng/μL]	1127.0	1441.0	983.0	643.0
	rRNA Ratio [28S/18S]	1.4	1.4	0.6	0.7
18S	Start size [nt]	1609.0	1590.0	1644.0	1616.0
	End size [nt]	2193.0	2196.0	1973.0	1951.0
	Area [%]	147.2	181.8	15.7	14.8
	% of total area	10.2	9.9	1.3	1.8
28S	Start size [nt]	2615.0	2588.0	2748.0	2653.0
	End size [nt]	3555.0	3559.0	3432.0	3293.0
	Area [%]	213.3	257.2	9.5	9.7
	% of total area	14.8	14.0	0.8	1.2

**Table 2:** RNA, mRNA and library concentration (Rc—control samples with high viability, Lv—long-term stored samples with low viability; each sample represented by two biological replicates, A —cDNA synthesis path for samples with RIN > 3, B— cDNA synthesis path for samples with low RIN < 3).

Sample ID	Rc 1A	Rc 2A	Lv 1B	Lv 2B	Lv 1A	Lv 2A
RNA concentration after isolation [ng/µL] *	2103.51	2148.00	1932.05	1256.31	1932.05	1256.31
RNA concentration after DNA removal [ng/µL]*	900.40	901.56	906.50	904.28	906.50	904.28
mRNA fraction concentration [ng/µL] *	126.72	124.56	90.61	62.88	90.61	62.88
library concentration [ng/µL] **	11.1	11.31	0.76	0.82	5.38	5.4

* measured by NanoDrop 1000, ** measured by Qubit.

**Table 3:** Qualitative report of RNA-Seq results from QC report analysis.

Samples	Rc 1A	Rc 2A	Lv 1A	Lv 2A
**Forward**				
Number of reads	37,648,549	38,165,239	28,512,788	36,711,845
Number of bases	5,061,029,534	4,977,869,871	3,498,639,578	5,067,910,888
Read length	15–150	15–150	15–150	15–150
%GC	55	55	55	55
**Reverse**				
Number of reads	37,648,549	38,165,239	28,512,788	36,711,845
Number of bases	5,012,350,442	4,932,157,953	3,466,518,530	5,013,123,377
Read length	15–150	15–150	15–150	15–150
%GC	54	54	54	54

## Supplementary Materials

The following are available online at https://www.mdpi.com/2073-4425/11/10/1190/s1, Figure S1: Mean sequencing quality across all bases; Figure S2: Mean quality score per read distribution; Figure S3: The content of each type of base; Figure S4: Combined content of G and C bases; Figure S5: Combined content of ambiguous bases; Figure S6: Sequence Length Distribution. Table S1: Concentration of fraction mRNA in library.
