# Supplementary material for: Low RIN Value for RNA-Seq Library Construction from Long-Term Stored Seeds: A Case Study of Barley Seeds
Source: Genes (Basel). 2020 Oct 13;11(10):1190. doi: 10.3390/genes11101190 (PMC7650657; doi:10.3390/genes11101190)

Qualitative report of RNA-Seq results from QC report analysis

1. Per base sequence quality

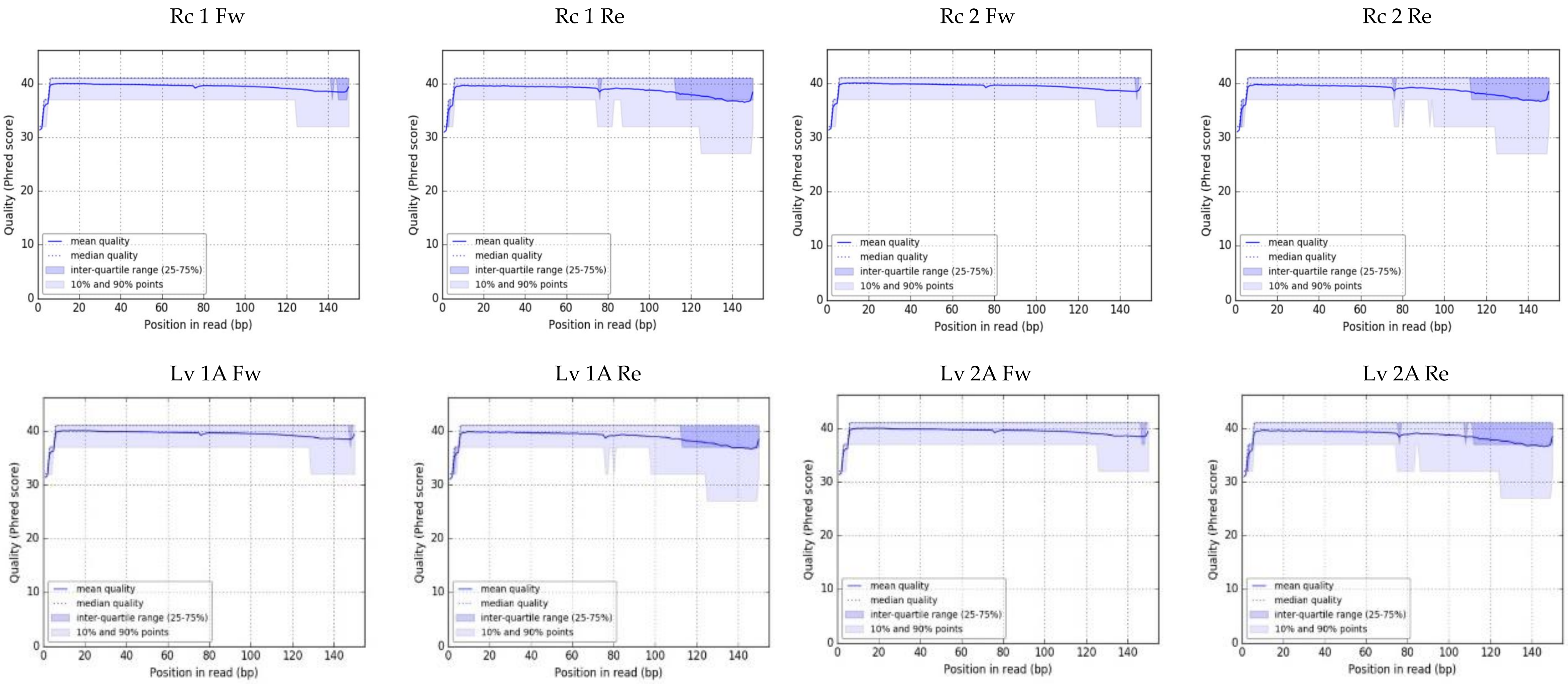

Figure 1: Mean sequencing quality across all bases

2. Per sequence quality scores

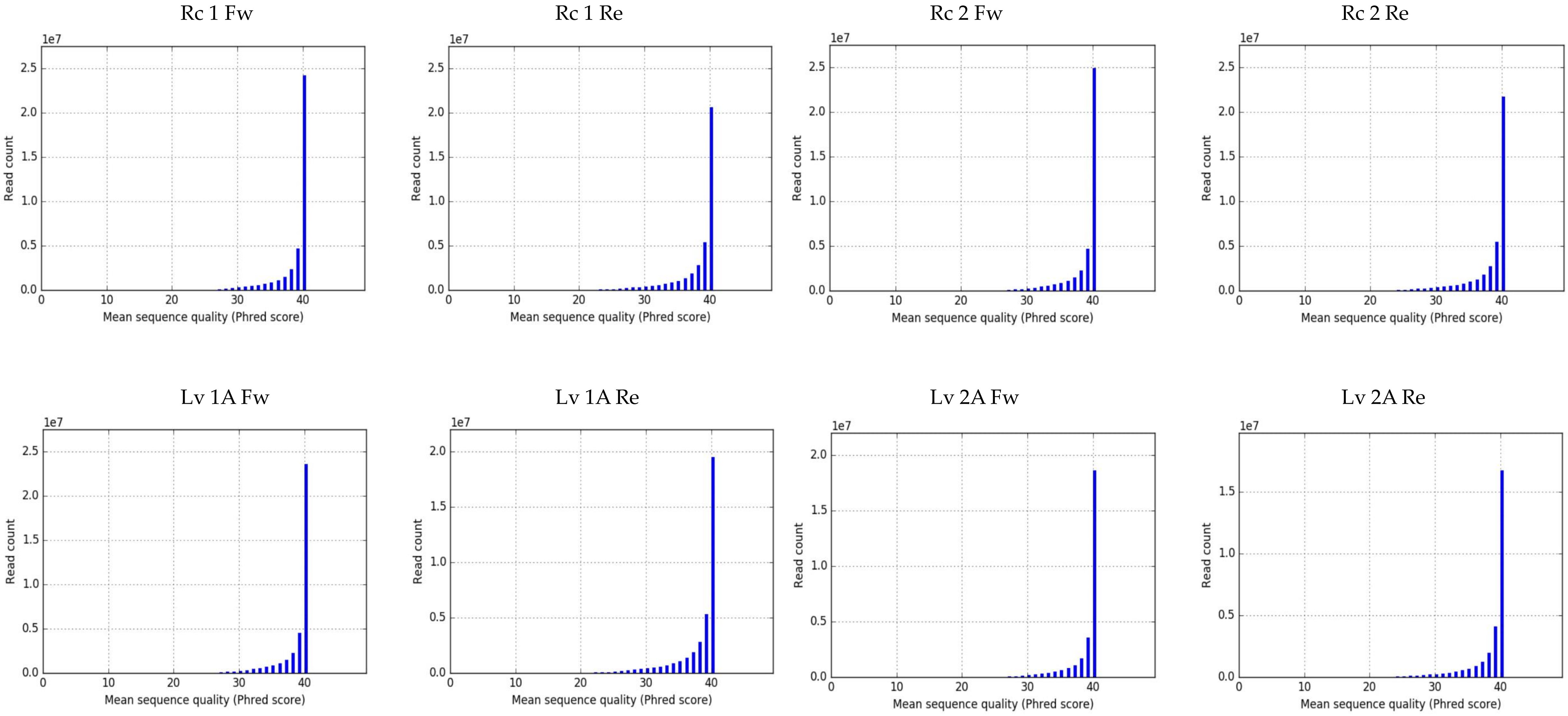

Figure 2: Mean quality score per read distribution

3. Per base sequence content

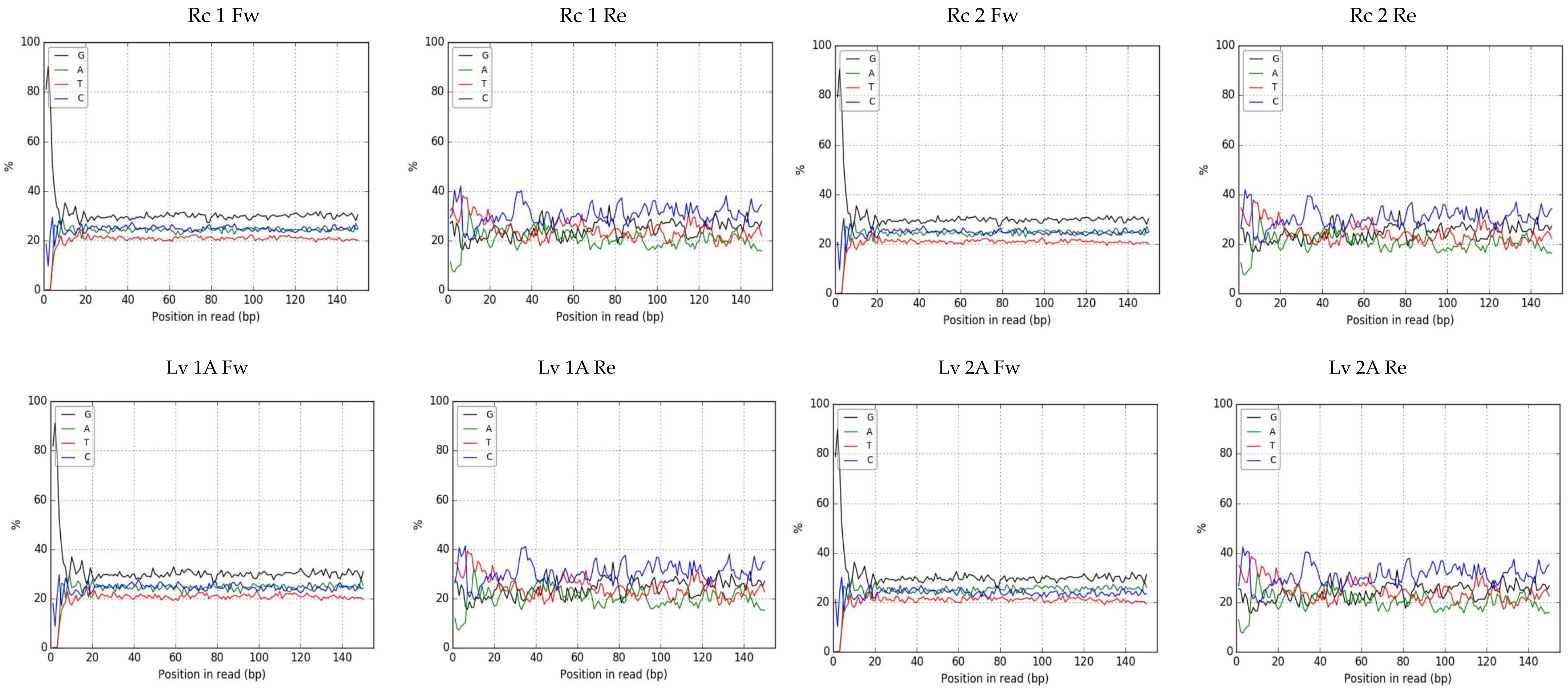

Figure 3: The content of each type of base

4. Per sequence GC content

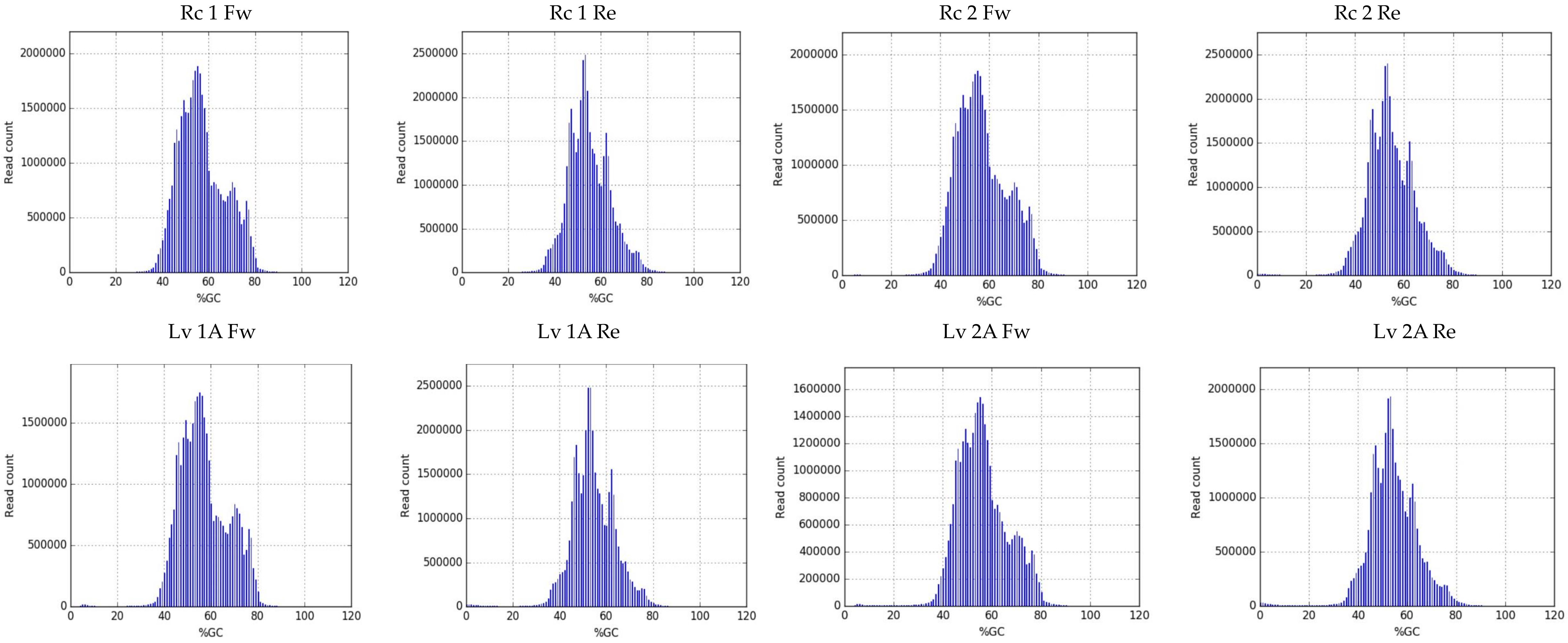

Figure 4: Combined content of G and C bases.

5. Ambiguous base content

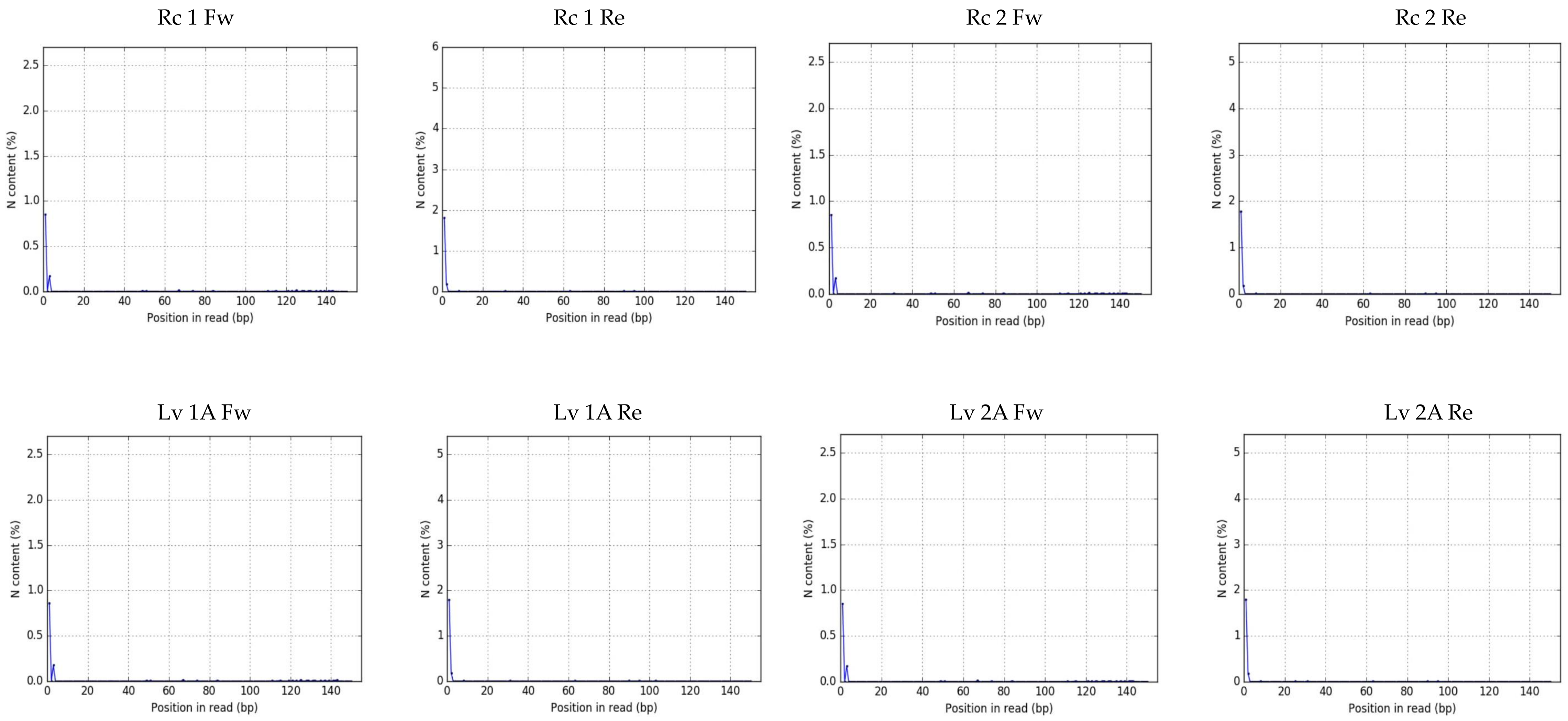

Figure 5: Combined content of ambiguous bases.

6. Sequence Length Distribution

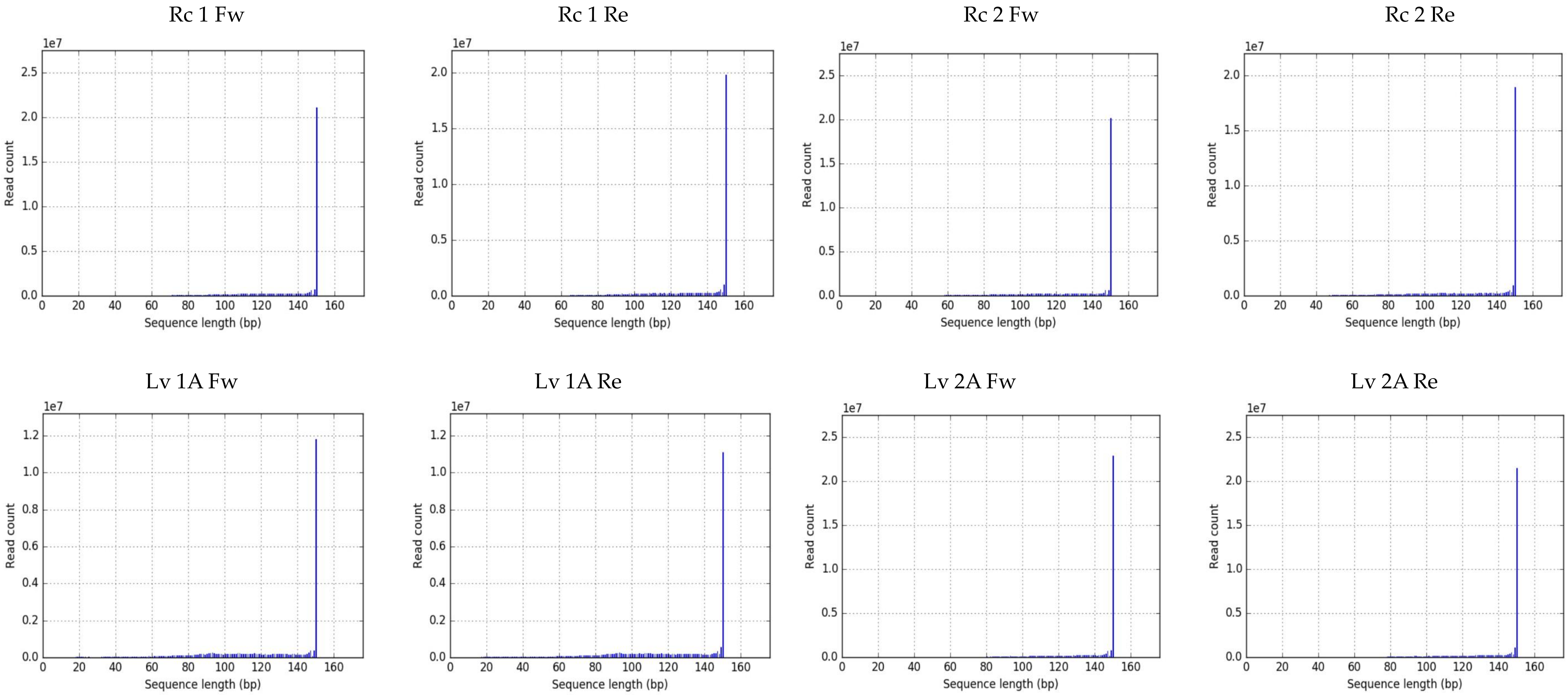

Supplement: Supplementary file 1 [file genes-11-01190-s001.zip › Figure S1.pdf]
