# Supplementary material for: Low RIN Value for RNA-Seq Library Construction from Long-Term Stored Seeds: A Case Study of Barley Seeds
Source: Genes (Basel). 2020 Oct 13;11(10):1190. doi: 10.3390/genes11101190 (PMC7650657; doi:10.3390/genes11101190)
Supplement: Supplementary file 1 [file genes-11-01190-s001.zip › Table S1.docx]

Table S1. Concentration of fraction mRNA in library

| Rc-1 | | | Rc-2 | | | Lv-1 b | | | Lv-2 b | | | Lv-1 c | | | Lv-2 c | | |
| --- | --- | --- | --- | --- | --- | --- | --- | --- | --- | --- | --- | --- | --- | --- | --- | --- | --- |
| Size [bp] | Conc. [pg/ml] | Molarity [pmol/l] | Size [bp] | Conc. [pg/ml] | Molarity [pmol/l] | Size [bp] | Conc. [pg/ml] | Molarity [pmol/l] | Size [bp] | Conc. [pg/ml] | Molarity [pmol/l] | Size [bp] | Conc. [pg/ml] | Molarity [pmol/l] | Size [bp] | Conc. [pg/ml] | Molarity [pmol/l] |
| 69 | 15.34 | 338.30 | 69 | 12.71 | 280.70 | 83 | 9.50 | 172.50 | 444 | 7.19 | 24.50 | 88 | 34.52 | 594.00 | 88 | 41.07 | 707.00 |
| 92 | 172.08 | 2 839.50 | 88 | 59.21 | 1 024.30 | 347 | 8.09 | 35.30 | 458 | 13.35 | 44.20 | 94 | 64.50 | 1 043.70 | 94 | 75.36 | 1 212.50 |
| 259 | 2 038.59 | 11 926.20 | 93 | 91.14 | 1 482.80 | 391 | 6.05 | 23.50 | 492 | 20.27 | 62.50 | 288 | 2 138.73 | 11 266.90 | 286 | 1 436.54 | 7 600.90 |
| 274 | 858.42 | 4 746.70 | 259 | 1 906.78 | 11.145.8 | 403 | 5.11 | 19.20 | 537 | 65.34 | 184.40 | 336 | 132.86 | 599.20 | 290 | 1 132.45 | 5 912.60 |
| 288 | 1 558.88 | 8 187.10 | 274 | 705.30 | 3 904.80 | 421 | 4.15 | 14.90 | 574 | 13.39 | 35.40 | 349 | 376.64 | 1 637.40 | 337 | 198.69 | 893.00 |
| 313 | 766.70 | 3 707.30 | 285 | 1 516.68 | 8 050.20 | 439 | 6.32 | 21.80 | 623 | 20.31 | 49.40 | 370 | 200.34 | 820.00 | 349 | 336.70 | 1 462.90 |
| 350 | 763.81 | 3 304.90 | 316 | 618.53 | 2 968.00 | 454 | 17.11 | 57.10 | 668 | 9.68 | 21.90 | 392 | 92.18 | 355.90 | 372 | 252.83 | 1 030.10 |
| 369 | 453.24 | 1 861.70 | 336 | 267.77 | 1 207.00 | 488 | 24.71 | 76.70 | 755 | 14.32 | 28.70 | 397 | 206.37 | 787.60 | 396 | 338.56 | 1 296.60 |
| 395 | 598.91 | 2 297.70 | 349 | 577.11 | 2 508.50 | 514 | 7.65 | 22.60 | 887 | 130.23 | 222.50 | 428 | 217.39 | 769.10 | 432 | 199.56 | 700.50 |
| 430 | 413.21 | 1 456.50 | 371 | 429.58 | 1 756.00 | 538 | 52.84 | 148.80 | 1 267 | 86.34 | 103.20 | 477 | 162.60 | 516.80 | 480 | 100.24 | 316.40 |
| 476 | 349.86 | 1 114.40 | 394 | 539.45 | 2 075.30 | 570 | 12.86 | 34.20 | 1 489 | 62.84 | 63.90 | 542 | 53.42 | 149.40 | 504 | 50.21 | 151.10 |
| 532 | 93.41 | 266.00 | 428 | 398.00 | 1 407.90 | 624 | 19.42 | 47.20 |  |  |  | 568 | 15.45 | 41.20 | 538 | 42.45 | 119.50 |
| 566 | 33.05 | 88.50 | 478 | 209.36 | 663.70 | 667 | 6.96 | 15.80 |  |  |  | 583 | 71.18 | 184.90 | 563 | 16.74 | 45.10 |
| 584 | 108.57 | 281.60 | 504 | 111.39 | 334.80 | 880 | 115.08 | 198.20 |  |  |  | 733 | 6.77 | 14.00 | 584 | 32.13 | 83.30 |
|  |  |  | 533 | 154.48 | 439.10 | 1 266 | 67.23 | 80.50 |  |  |  | 841 | 5.58 | 10.10 | 6 775 | 4.07 | 0.90 |
|  |  |  | 596 | 75.98 | 193.20 | 1 498 | 45.61 | 46.10 |  |  |  | 1 051 | 16.46 | 23.70 | 7 728 | 4.10 | 0.80 |
|  |  |  | 710 | 10.82 | 23.10 | 1 970 | 10.01 | 7.70 |  |  |  | 1 287 | 6.78 | 8.00 |  |  |  |
|  |  |  |  |  |  | 2 524 | 3.59 | 2.20 |  |  |  | 1 404 | 6.38 | 6.90 |  |  |  |
|  |  |  |  |  |  | 2 821 | 1.66 | 0.90 |  |  |  | 2 086 | 4.42 | 3.20 |  |  |  |
|  |  |  |  |  |  |  |  |  |  |  |  | 2 932 | 2.30 | 1.20 |  |  |  |
|  |  |  |  |  |  |  |  |  |  |  |  | 5 560 | 6.11 | 1.70 |  |  |  |
|  |  |  |  |  |  |  |  |  |  |  |  | 6 151 | 5.40 | 1.30 |  |  |  |
|  |  |  |  |  |  |  |  |  |  |  |  | 6 778 | 4.35 | 1.00 |  |  |  |
|  |  |  |  |  |  |  |  |  |  |  |  | 7 307 | 6.05 | 1.30 |  |  |  |
